# Endogenous Acute Phase Serum Amyloid A Lacks Pro-Inflammatory Activity, Contrasting the Two Recombinant Variants That Activate Human Neutrophils through Different Receptors

**DOI:** 10.3389/fimmu.2013.00092

**Published:** 2013-04-22

**Authors:** Karin Christenson, Lena Björkman, Sofie Ahlin, Maja Olsson, Kajsa Sjöholm, Anna Karlsson, Johan Bylund

**Affiliations:** ^1^The Phagocyte Research Laboratory, Department of Rheumatology and Inflammation Research, EULAR Centre of Excellence in Rheumatology, Sahlgrenska Academy, University of GothenburgGothenburg, Sweden; ^2^Sahlgrenska Center for Cardiovascular and Metabolic Research, Sahlgrenska Academy, University of GothenburgGothenburg, Sweden

**Keywords:** inflammation, neutrophils, acute phase proteins, arthritis

## Abstract

Most notable among the acute phase proteins is serum amyloid A (SAA), levels of which can increase 1000-fold during infections, aseptic inflammation, and/or trauma. Chronically elevated SAA levels are associated with a wide variety of pathological conditions, including obesity and rheumatic diseases. Using a recombinant hybrid of the two human SAA isoforms (SAA1 and 2) that does not exist *in vivo*, numerous *in vitro* studies have given rise to the notion that acute phase SAA is a pro-inflammatory molecule with cytokine-like properties. It is however unclear whether endogenous acute phase SAA *per se* mediates pro-inflammatory effects. We tested this in samples from patients with inflammatory arthritis and in a transgenic mouse model that expresses human SAA1. Endogenous human SAA did not drive production of pro-inflammatory IL-8/KC in either of these settings. Human neutrophils derived from arthritis patients displayed no signs of activation, despite being exposed to severely elevated SAA levels in circulation, and SAA-rich sera also failed to activate cells *in vitro*. In contrast, two recombinant SAA variants (the hybrid SAA and SAA1) both activated human neutrophils, inducing L-selectin shedding, production of reactive oxygen species, and production of IL-8. The hybrid SAA was approximately 100-fold more potent than recombinant SAA1. Recombinant hybrid SAA and SAA1 activated neutrophils through different receptors, with recombinant SAA1 being a ligand for formyl peptide receptor 2 (FPR2). We conclude that even though recombinant SAAs can be valuable tools for studying neutrophil activation, they do not reflect the nature of the endogenous protein.

## Introduction

Serum amyloid A (SAA) is one of the most prominent acute phase proteins in the body and the levels in plasma can rise 1000-fold in response to inflammation, infection, or tissue injury. The SAAs comprise a family of apolipoproteins with characteristic structural features (Uhlar and Whitehead, 1999). In humans, four distinct SAA genes exist, *SAA1-SAA4*. The *SAA3* is not expressed at all (Sellar et al., 1994) while *SAA4* is constitutively expressed. The remaining two isoforms, SAA1 and SAA2, are the acute phase variants of SAA and are mainly produced by hepatocytes (Uhlar and Whitehead, 1999), although extrahepatic SAA production can also occur (Upragarin et al., 2005). In obesity, a condition characterized by low-grade inflammation with moderately elevated levels of acute phase proteins (Smorlesi et al., 2012), adipose tissue is the major source of SAA (Sjoholm et al., 2005). Acute phase SAA is the precursor of reactive amyloidosis which can follow after long-standing inflammatory conditions, e.g., rheumatic diseases. During reactive amyloidosis, deposits of amyloid A form amyloid fibrils in tissue, leading to progressive organ failure (Benson et al., 2006). In line with this, SAA has been shown to serve as a reliable biomarker for the assessment of inflammatory joint disease and it has been suggested that SAA is directly involved in the pathogenesis of inflammatory arthritis (Cunnane et al., 2000; Connolly et al., 2012).

As for the function of acute phase SAA in the context of immunology, the endogenous protein has been elegantly shown to serve as an innate recognition molecule that opsonizes Gram-negative bacteria by binding to Outer membrane protein A (Shah et al., 2006). Based on *in vitro* studies employing a recombinant protein (described below), SAA is also believed to possess a variety of directly pro-inflammatory properties with cytokine-like features; most notably it has been described as a chemoattractant for phagocytic cells and to induce production of pro-inflammatory cytokines such as IL-8 (Badolato et al., 2000; Furlaneto and Campa, 2000; Hatanaka et al., 2004; Bjorkman et al., 2008; He et al., 2008; Lee et al., 2008). In line with this, a recent paper describes the existence of activated neutrophils in circulation of melanoma patients (De Santo et al., 2010) where the authors conclude that high levels of endogenous plasma SAA are responsible for the cell activation.

The pro-inflammatory effects of SAA have mainly been dissected by the use of a commercially available recombinant form of SAA (Badolato et al., 2000; He et al., 2003, 2008; Bjorkman et al., 2008; Lee et al., 2008). This recombinant SAA hybrid (rSAAh) is a chimeric molecule corresponding to the sequence of human SAA1 with an additional N-terminal methionine and two mutated amino acids corresponding to the sequence of SAA2. In terms of receptor preference for mediating its pro-inflammatory effects, rSAAh has been reported to be a ligand of a variety of receptors, including Toll-like receptor 2 (Cheng et al., 2008) and the scavenger receptors CLA-1 (Baranova et al., 2005) and CD36 (Baranova et al., 2010). In addition, the chemoattractant receptor formyl peptide receptor 2 (FPR2; formerly FPRL1) has been shown to be the preferred receptor for many SAA-induced effects on phagocytes (Su et al., 1999; He et al., 2003; Lee et al., 2008).

The design of the rSAAh is based on genetic variation within the *SAA1* and *SAA2* genes. Each of the individual substitutions of rSAAh has been described as naturally occurring variants of SAA1 (Baba et al., 1992). An allelic variant identical to the full sequence of rSAAh has however to our knowledge not been described. Thus, the rSAAh protein is not identical to any of the reported human SAA isoforms and we have previously demonstrated that this recombinant protein is functionally different from the endogenous acute phase SAA present in circulation during severe inflammation. Using purified endogenous acute phase SAA, as well as blood samples from rheumatic patients with elevated levels of endogenous SAA, we found the endogenous SAA to be remarkably inert, whereas rSAAh potently activated human phagocytes (Bjorkman et al., 2010). Despite these discrepancies with regards to sequence and function, rSAAh continues to be used as a substitute for the native molecule.

Recently, a recombinant SAA1 (rSAA1) was launched with an amino acid sequence identical to the endogenous human SAA1. This inspired us to further investigate the role of SAA1 in inflammatory events, and to discern whether this molecule can better represent the endogenous protein than the rSAAh. We first found equal plasma levels of IL-8 between healthy controls and patients with severely elevated SAA levels due to inflammatory arthritis, indicating that native acute phase SAA does not drive the production of this pro-inflammatory cytokine. We also employed a transgenic mouse model with circulating levels of SAA similar to the levels seen in human obesity (Olsson et al., 2011). Also in this setting, we found that plasma levels of murine IL-8 (KC), were not higher in transgenic than in wild type (WT) mice. Both recombinant forms of SAA activated pro-inflammatory mechanisms in human neutrophils, in striking contrast to the effects induced by SAA-rich patient plasma, which failed to activate neutrophils *in vitro* as well as *in vivo*. The rSAA1-induced effects were mediated by FPR2, whereas this receptor did not mediate neutrophil responses to rSAAh. We conclude that although the recombinant SAA proteins can be useful tools for studies of neutrophil activation, they are invalid substitutes for endogenous acute phase SAA.

## Patients and Methods

### Reagents

HRP was purchased from Boehringer-Mannheim (Mannheim, Germany) and LPS from *Escherichia coli* (serotype O111:B4), Isoluminol, and TNF were from Sigma Chemical Co. (St. Louis, MO, USA). Dextran and Ficoll-Paque were from Pharmacia (Uppsala, Sweden). The FPR2 antagonist WRWWWW (WRW4) was from GenScript Corp. (Piscataway, NJ, USA) and acetoxymethylated Fura-2 (Fura-2AM) from Molecular Probes Inc. (Eugene, OR, USA). Anti-CD62L (L-selectin) was from BD Biosciences (Stockholm, Sweden). Cyclosporin H was provided by Novartis Pharma (Basel, Switzerland). Annexin V FLUOS was from Roche Diagnostics (Mannheim, Germany) and 7-amino-actinomycin D (7-AAD) from BD Biosciences (Stockholm, Sweden).

**Figure 1:**
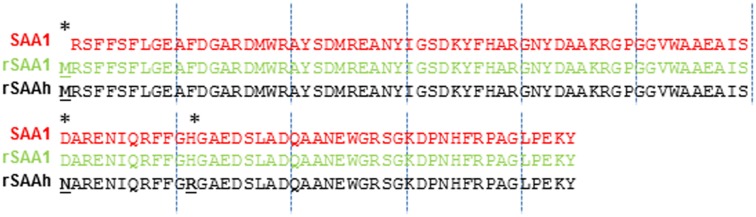
**Amino acid sequences of endogenous SAA1, rSAA1, and rSAAh**. The rSAAh is a hybrid molecule corresponding mainly to human SAA1 with the exception of the presence of an N-terminal methionine and two substitutions (derived from the sequence of SAA2): asparagine for aspartic acid at position 60 and arginine for histidine at position 71 (marked by asterisks). Recombinant SAA1 is identical to endogenous SAA1 except for the presence of an N-terminal methionine.

The recombinant SAA proteins were from PeproTech Inc. (Rocky Hill, NJ, USA) and had both endotoxin levels <0.1 ng/μg (1 EU/μg). Recombinant Human Apo-SAA (with amino acid sequence, cat. no 300-13; referred to in the text as rSAAh), is a hybrid molecule between sequences for SAA1 and SAA2 (two substitutions are derived from the sequence of SAA2: asparagine for aspartic acid at position 60 and arginine for histidine at position 71). The sequence for recombinant Human Apo-SAA1, cat. no 300-53; referred to in the text as rSAA1) is solely based on the sequence for SAA1. Both recombinant proteins reportedly contain an N-terminal methionine residue not present in the human SAA sequences (Figure 1).

### Patients

Peripheral blood was obtained from patients in flares of various forms of inflammatory arthritis (Table 1). All patients gave written informed consent. The study was approved by the Regional Ethics Board (No S010-03).

**Table 1:** **Patients included in the study**.

Patient no.	SAA in plasma μM (mg/l)	Diagnosis^a^	Treatment^b^	Gender
1	15 (180)	RA	MTX	Female
2	73 (855)	RA	SZS, pred.	Male
3	49 (570)	SpA/Am	pred.	Male
4	3 (32)	RA	MTX, pred.	Male
5	78 (910)	PsA	pred.	Male
6	428 (5000)	RA	MTX, pred. SZS, NSAIDs	Female
7	368 (4300)	Wg	methpred.	Male
8	12 (140)	SpA	MTX, α-TNF	Male
9	197 (2300)	RA	MTX, NSAIDs	Male

*^a^RA, rheumatoid arthritis; SpA/Am, spondyloarthritis/Amyloidosis; PsA, psoriasis arthritis; Wg, Wegeners granulomatosis*.

*^b^MTX, Methotrexate; pred., prednisolone; SZS, Sulfasalazine; NSAIDs, non-steroidal anti-inflammatory drugs; methpred., methylprednisolone*.

### Transgenic mouse model with adipose tissue expression of human SAA1

Generation of a transgenic mouse model with over-expression of human SAA1 in the adipose tissue has previously been described (Olsson et al., 2011). Levels of human SAA were measured in plasma from the hSAA transgenic animals and four WT animals using the Human SAA ELISA kit (Biosource, Camarillo, CA, USA). Undiluted plasma from WT or transgene mice were subjected to a mouse CXCL1/KC DuoSet ELISA development kit (R&D Systems, Minneapolis, MN, USA) according to the manufacturer’s description.

All animal study protocols were approved by the local Ethics Committee for animal studies at the administrative court of Appeals in Gothenburg, Sweden, approval numbers 201-2006 and 281-2008.

### Isolation of human neutrophils and preparation of plasma

Blood was collected in heparinized tubes and neutrophils were either analyzed directly after lysis of erythrocytes concomitant with fixation by lysis buffer (BD Biosciences, Sweden) or after purification according to Boyum et al. (1991) Neutrophils from buffy coats were isolated by Ficoll-Pacque gradient centrifugation. Purified neutrophils were resuspended in Krebs-Ringer phosphate buffer containing glucose (KRG) supplemented with 1 mM Ca^2+^ and stored on melting ice until use. Blood samples obtained for preparation of plasma was centrifuged for 10 min at 400 × *g* in room temperature, after which plasma was removed and stored at −70°C. Plasma from patients and controls were analyzed for SAA content using a hSAA ELISA Kit from Invitrogen (Camarillo, CA, USA; upper detection limit 600 mg/l and lower limit 11 mg/l) by the Clinical Immunological Laboratory (Sahlgrenska University Hospital). Plasma SAA of <11 mg/l, corresponding to 1 μM, are considered normal.

### NADPH-oxidase activity and priming

Neutrophil NADPH-oxidase activity was determined using an isoluminol-enhanced chemiluminescence technique, which measures the release of superoxide anion, the primary oxygen metabolite generated by the assembled NADPH-oxidase (Segal et al., 2012). The chemiluminescence activity was measured as described in Dahlgren et al. (2007). Where indicated, neutrophils were incubated at 37°C for 20 min in the presence of priming agent (TNF; 10 ng/ml), before addition of stimulus (50 μl). Priming with TNF-α induces degranulation with accompanying upregulation of receptors that maximizes subsequent ROS release triggered by chemoattractants (Bjorkman et al., 2008). When the FPR2 antagonist WRW4 was used, this compound was added 5 min before stimulation with SAA and the NADPH-oxidase activity was continuously recorded.

### Measurement of intracellular Ca^2+^

Neutrophils (2 × 10^7^/ml) were labeled with Fura-2AM (2 μM) in calcium-free KRG with BSA (0.1%) at room temperature for 30 min and were from there on protected from light. For laboratory procedure and details see reference (Karlsson et al., 2009) The intracellular Ca^2+^ levels are plotted as the fluorescence ratio (340/380 nm) over time.

### Neutrophil culture

Purified neutrophils (5 × 10^6^/ml) were incubated (5% CO_2_, 37°C) for 20 min in complete medium [RPMI 1640 supplemented with 10% FCS and 1% penicillin/streptomycin (PEST)] or alternatively in plasma (100%) from patients or controls. After addition of rSAAh, rSAA1, or LPS at designated concentrations, the cells were incubated for 20 h.

### Assessment of apoptosis

Evaluation of cell death was performed essentially as described in Christenson et al. (2012). In short, cells were stained with Annexin V in combination with 7-AAD and analyzed by flow cytometry on an Accuri C6 (Becton Dickinson, Mountain View, CA, USA). Data were analyzed using CFlow plus software.

### Determination of L-selectin expression

The exposure of CD62L (L-selectin) on neutrophils was assessed in a whole blood system (Bjorkman et al., 2010) by immunostaining and FACS-analysis. All data were analyzed using WinMDI 2.8 software or CFlow Plus.

### Quantification of intracellular IL-8, total IL-8 from cell culture, and cytokines in plasma samples

Freshly prepared neutrophils (10^6^/sample) from patients and controls were, after fixation and permeabilization (BD Cytofix/Cytoperm Plus, BD, San Diego, CA, USA), labeled with an allophycocyanin-conjugated anti-IL-8 Ab or an isotype control. Intracellular IL-8 was thereafter evaluated by flow cytometry on the basis of allophycocyanin fluorescence using an Accuri C6 (BD, USA) and analyzed by CFlowPlus. For the quantification of total IL-8 production after *in vitro* culture, Triton-X 100 (0.1%), and the protease inhibitor Pefa-Bloc (1 mM) was added to cell cultures and lysates were analyzed by a human IL-8 DuoSet ELISA development kit (R&D Systems, Minneapolis, MN, USA) according to the manufacturer’s description. Plasma (60 μl, diluted 1:3) from all patients were analyzed for IL-1β, IL6, IL-8, IL-10, IL-12, GM-CSF, TNF, INF-γ, and IP-10 using a Bio-plex Pro Cytokine assay (Bio-Rad Laboratories AB, Sundbyberg, Sweden) according to the manufacturer’s instructions.

## Results

### Human SAA does not drive production of pro-inflammatory IL-8 in patients with inflammatory arthritis or of KC in a murine model with transgenic adipose tissue expression of human SAA1

**Figure 2:**
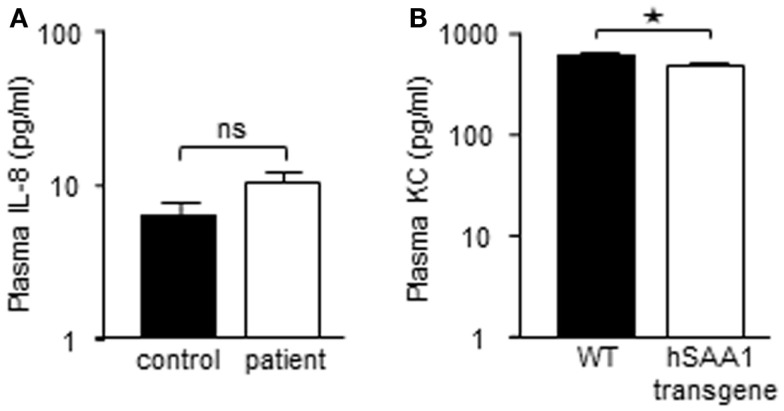
**Elevated levels of SAA is not associated with heightened expression of IL-8 in circulation of rheumatic patients, or of KC in a mouse model of diet-induced obesity**. **(A)** Cell-free plasma from nine patients with inflammatory arthritis (P#1-#9; white bar) and three healthy controls (black bar) were evaluated for IL-8 content using a Luminex analysis; shown is mean (+SEM). **(B)** WT and transgenic mice expressing human SAA1 in adipocytes were fed a high-fat diet to induce obesity. Obese transgenic mice have circulating levels of human SAA1 similar to the levels seen in human obesity. Plasma samples were analyzed by ELISA for the murine IL-8 counterpart CXCL1/KC and are shown as mean (+SEM; WT mice; *n* = 10 black bar, transgenic mice; *n* = 8, white bar). Statistical analyses were performed using unpaired *t*-tests (*p < 0. 05; ns, not significant).

**Figure 3:**
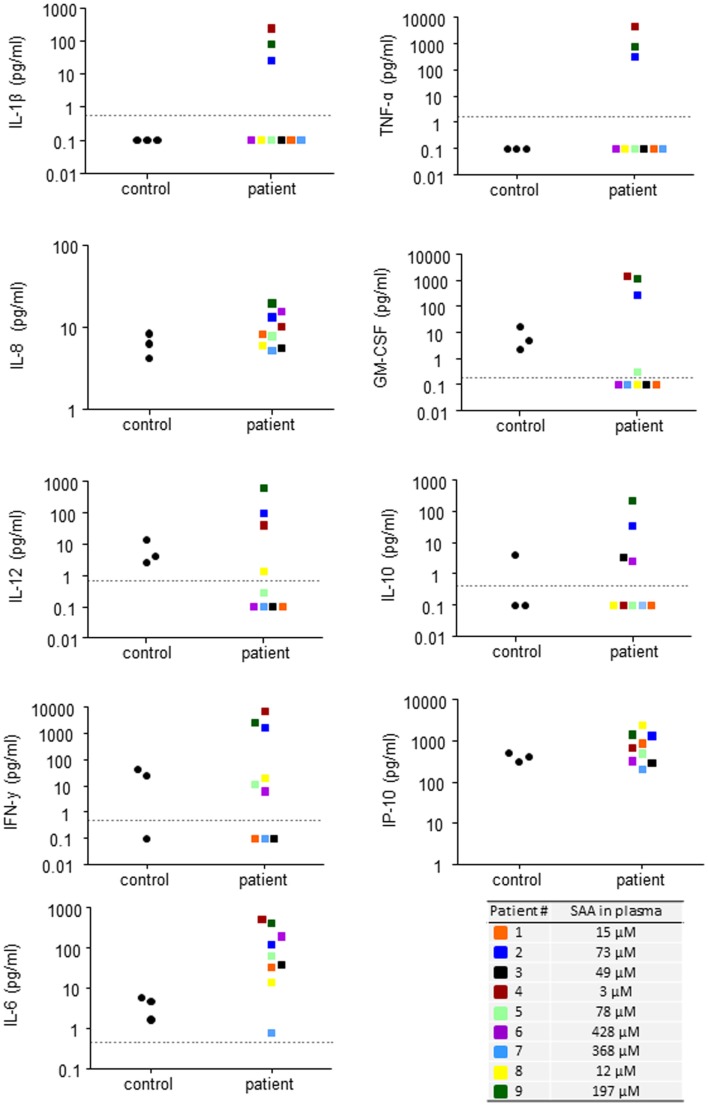
**High SAA levels in blood do not correspond to increased levels of inflammatory cytokines**. Plasma levels of inflammatory cytokines in the individual patients included in the study. Dotted lines represent the detection limits for each cytokine; samples lower than detection limit were given a value of 0.1 pg/ml to facilitate presentation of data. Circulating SAA levels for each patient are shown (additional data in Table 1).

Recombinant hybrid SAA (rSAAh) potently induce the activation of NF-κB in leukocytes, resulting in the production and release of pro-inflammatory cytokines, e.g., IL-8 (He et al., 2008; Okamoto et al., 2008). To study potential pro-inflammatory effects of native SAA we enrolled nine patients with inflammatory arthritis and circulating SAA levels varying between 3 and >400 μM (Table 1). Analyzing pro-inflammatory cytokines in the plasma from these patients, we found the levels of IL-8 to be similar to that of healthy controls (Figure 2A). For a number of other cytokines we found that, although some were elevated in the patient group as a whole, there was a remarkable lack of correlation between SAA concentrations and levels of inflammatory cytokines in patient plasma (Figure 3).

Also in murine systems rSAAh has been shown to drive inflammatory signaling (Niemi et al., 2011). We next used an established transgenic mouse model (Olsson et al., 2011) where human *SAA1* is constitutively expressed in adipocytes. After the mice had been challenged with a high-fat diet, circulating levels of human SAA1 were similar to the levels seen in human obesity (3.07 ± 0.91 μM; mean ± SD, *n* = 8). The presence of human SAA1 in the transgenic mice did not seem to drive production of pro-inflammatory cytokines, but instead plasma levels of the murine IL-8 homolog KC were significantly lower in the transgenic mice than the WT mice (Figure 2B).

Taken together, these data imply that native human SAA does not drive the production of pro-inflammatory cytokines in circulation, neither in inflammatory arthritis patients, nor in a transgenic mouse model displaying moderately elevated levels of human SAA1.

### Recombinant but not endogenous SAA induce neutrophil IL-8 production

**Figure 4:**
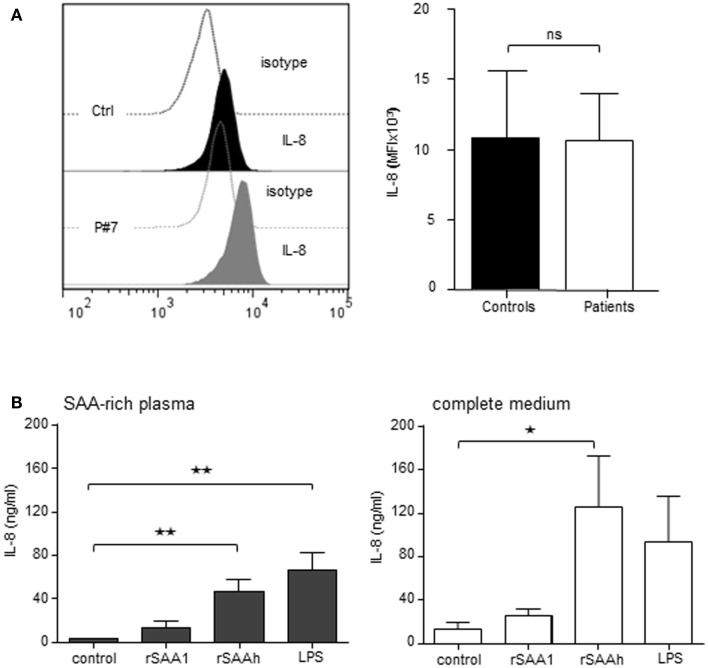
**Recombinant, but not endogenous, SAA induces IL-8 production in human neutrophils**. **(A)** Freshly prepared neutrophils were analyzed for intracellular IL-8 content using flow cytometry. The figures show representative histograms (P#7, left panel) and averages (+SEM) of the mean fluorescence intensity from three controls (black bar) and P#4-7 (white bar). **(B)** Freshly prepared patient neutrophils (P#1, #2, #3, #5, and #6) were incubated for 20 h in 100% endogenous plasma (left) or complete medium; RPMI + 10% FCS and 1% PEST (right) with or without addition of rSAA1 (10 μM), rSAAh (10 μM), or LPS (100 ng/ml). Total IL-8 content in the cultures was measured by ELISA and is shown as mean (+SEM). Statistical analyses were performed using unpaired students *t*-test **(A)** and One-way ANOVA followed by Dunnett‘s multiple comparison test **(B)**. **p < 0.01; ns = not significant.

In order to study if circulating endogenous SAA induces IL-8 production specifically in neutrophils, we analyzed freshly prepared cells from patients and healthy controls by intracellular immunostaining and flow cytometry. Patient neutrophils contained detectable IL-8, however, the levels were not higher than in neutrophils from healthy controls (Figure 4A). We next studied the effect of endogenous SAA on *de novo* synthesis of IL-8 by culturing purifird neutrophils for 20 h *in vitro* with or without SAA-rich patient plasma (ranging from 15 to 428 μM SAA; Table 1) or complete medium with 10% FCS. The levels of IL-8 were equal between cells having experienced SAA-rich plasma and cells incubated with control plasma (not shown). When spiking the SAA-rich plasma or complete medium with 10 μM of the rSAAh, a significant and robust IL-8 production was observed, in the same range as for SAA-rich plasma spiked with LPS (Figure 4B). The addition of recombinant SAA1 (rSAA1) to SAA-rich plasma or complete medium moderately increased IL-8 levels at this concentration (Figure 4B). These data show that SAA-rich plasma do not induce IL-8 production in neutrophils, while recombinant SAA does. Further, the SAA-rich patient plasma does not appear to contain factors that neutralize responses to recombinant SAA. Hence, the recombinant SAA proteins are functionally different from the endogenous protein in circulation of patients with inflammatory arthritis.

### Recombinant but not endogenous SAA shed neutrophil L-selectin

**Figure 5:**
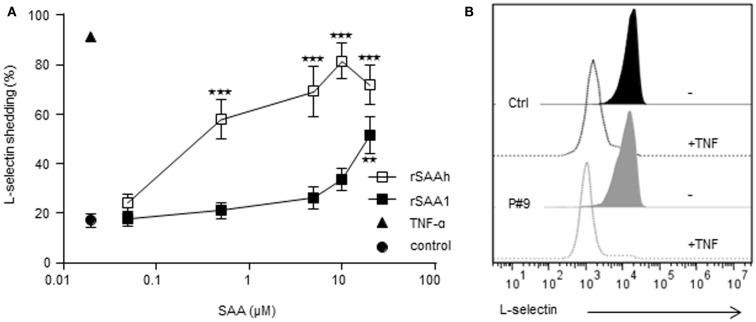
**Recombinant, but not endogenous, SAA activates neutrophils to shed L-selectin**. **(A)** Whole blood from healthy donors was stimulated *in vitro* as indicated for 20 min and surface expression of L-selectin was monitored by flow cytometry after gating on neutrophils on the basis of forward and side scatter. Stimulation with rSAAh (open squares) or rSAA1 (closed squares) induced L-selectin shedding in a dose-dependent manner. Data are shown as mean (±SEM, *n* = 3–6) and statistical analysis was performed using One-way ANOVA followed by Dunnett‘s multiple comparison test where the SAA-treated samples were compared to the unstimulated control (**p < 0.01; ***p < 0.001). Stimulation with the positive control TNF (10 ng/ml; closed triangle) is shown for comparison. **(B)** Freshly drawn blood from P#9 (gray; 197 μM SAA in plasma) and one healthy control (black) were analyzed for L-selectin expression after treatment with (closed histograms) or without (open histograms) TNF-α (10 ng/ml). Histograms are representatives of numerous patients assayed independently, consistently showing resting levels of L-selectin that was efficiently shed by treatment with TNF.

To further explore potential neutrophil activating properties of endogenous SAA, we monitored L-selectin expression. This adhesion molecule is present on resting cells, but is rapidly cleaved off from the cell surface during cell activation (Ley et al., 2007). Whole blood samples from healthy controls were stimulated with the recombinant forms of SAA for 20 min after which the surface expression of L-selectin was measured by flow cytometry. TNF was used as a positive control, inducing an activated neutrophil phenotype devoid of surface L-selectin (Figure 5). Both recombinant forms of SAA-induced L-selectin shedding in a dose-dependent manner (Figure 5A). The rSAAh was more potent than rSAA1, with an EC_50_ value 100-fold lower, but at 20 μM both recombinant forms of SAA significantly induced shedding of L-selectin (Figure 5A). In contrast, circulating neutrophils from patients with endogenous SAA levels far exceeding 20 μM displayed intact levels of surface L-selectin, shown in Figure 5B by a representative example (patient #9 with 197 μM SAA in plasma). This confirms our earlier report (Bjorkman et al., 2010) and we conclude that SAA-rich plasma from patients with rheumatic disease fails to activate neutrophils in contrast to the recombinant forms of SAA which both induce L-selectin shedding.

### The recombinant SAA variants differ in potency regarding neutrophil activation

**Figure 6:**
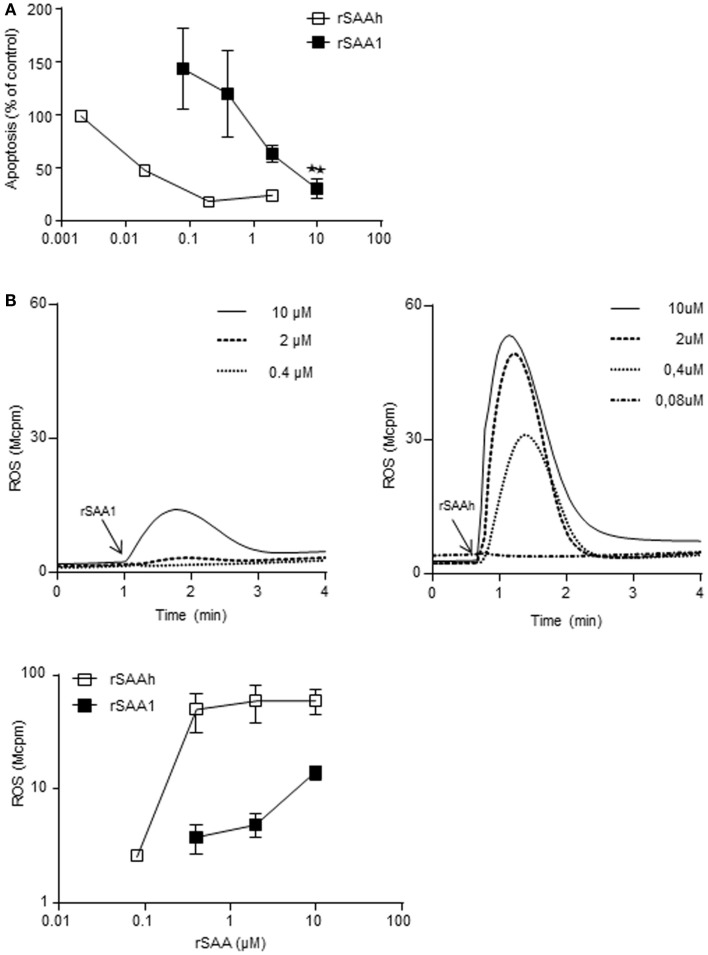
**The recombinant SAA proteins activate neutrophils with different potency**. **(A)** Neutrophils purified from buffy coats were incubated for 20 h with addition of medium, or rSAA1 (filled squares; *n* = 3) at the indicated concentrations. Apoptosis was thereafter evaluated by flow cytometry on the basis of Annexin V positive events. Shown for rSAA1 is mean (±SEM) and one representative experiment with rSAAh (open squares) is shown for comparison. Statistical analysis was performed using repeated measurement ANOVA followed by Dunnett‘s multiple comparison test (**p < 0.01). **(B)** Extracellular release of ROS from TNF-α primed neutrophils after addition (arrow) of indicated concentrations of rSAA1 or rSAAh. Representative curves (upper) are shown with arrows indicating the addition of SAA, as well as mean peak values (±SEM; lower) from three independent experiments.

Our results so far indicated that the two recombinant forms of SAA differ in potency regarding the induction of IL-8 production and L-selectin shedding, despite being very similar on the amino acid level. We investigated whether a similar pattern was evident also for other features of neutrophil activation. We have previously shown that the rSAAh dose-dependently delays neutrophil apoptosis at nanomolar concentrations (Christenson et al., 2008) and activates the NADPH-oxidase to produce reactive oxygen species (ROS) at higher doses (Bjorkman et al., 2008). For rSAA1 we also found a delayed neutrophil apoptosis, although with a potency that was approximately 100-fold lower than that of rSAAh (Figure 6A). A similar picture was evident for ROS production. Using TNF primed cells (in order to maximize responses to weak agonists), the highest tested concentration of rSAA1 (10 μM) resulted in low, but clearly measurable, levels of ROS release (Figure 6B). As compared to rSAAh, the potency for induction of ROS production was again approximately 100-fold lower (Figure 6B) Experimental limitations prohibited us from investigating rSAA1 at higher concentrations. We conclude that although rSAAh and rSAA1 induce activation of the same features in human neutrophils, the hybrid form is around 100-fold more potent than rSAA1.

### Recombinant SAA1 activates FPR2 on primary neutrophils

**Figure 7:**
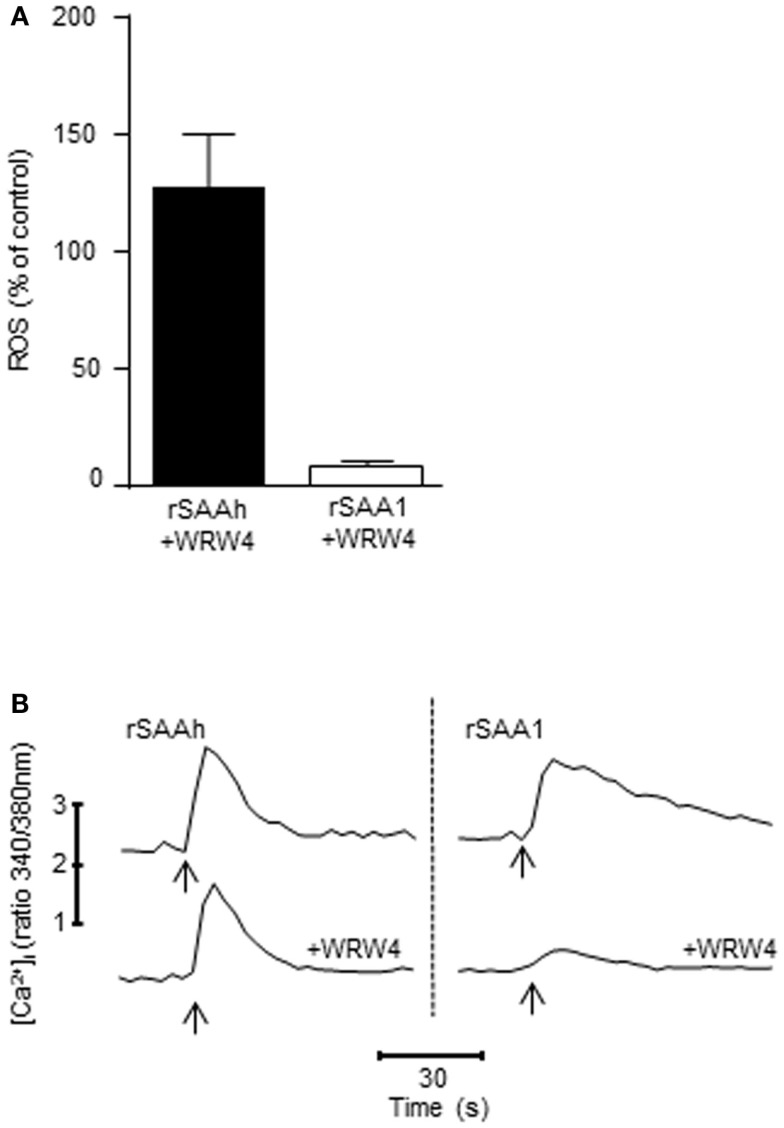
**FPR2 mediates ROS production and Ca^2+^ signaling induced by rSAA1, but not by rSAAh**. **(A)** The ROS release from TNF primed neutrophils was evaluated with a chemiluminescence assay after addition of rSAAh or rSAA1 (10 μM). When applicable, the FPR2-specific antagonist WRW4 (5 × 10^−6^ M) was added 5 min prior to stimulation. The bars show percent of control (no inhibitor) as mean (+SEM; *n* = 4) **(B)** Fura-2 labeled neutrophils were stimulated (arrows) with rSAAh (10 μM; left) or rSAA1 (10 μM; right) in the presence or absence of the FPR2-specific antagonist WRW4 (5 × 10^−6^ M). Traces depict intracellular Ca^2+^ levels plotted as the fluorescence ratio (340/380 nm) over time. The traces are representatives out of three to six independent experiments.

A wide variety of receptors have been claimed to recognize SAA, and among those is FPR2 (formerly known as formyl peptide receptor-like 1, FPRL1), a chemotactic receptor present on human phagocytes (Fu et al., 2006). We have previously demonstrated that even though FPR2 is capable of recognizing rSAAh in receptor-transfected cell lines, this receptor is not responsible for mediating rSAAh-induced ROS production in human neutrophils (Bjorkman et al., 2008). We utilized the FPR2-specific antagonist WRW4, at a concentration (5 × 10^−6^ M) where it does not interfere with signaling from other chemoattractant receptors (data not shown) and (Stenfeldt et al., 2007), and confirmed that ROS production induced by rSAAh is not mediated through FPR2 (Figure 7A). In contrast, the ROS production evoked by rSAA1 was completely inhibited by the FPR2 antagonist, indicating that FPR2 mediates the rSAA1-induced ROS release (Figure 7A). Neither rSAAh-, nor rSAA1-induced ROS production was inhibited by the specific FPR1 antagonist cyclosporin H (not shown).

The signal transduction from chemoattractant receptors to the NADPH-oxidase most often involves transient elevations of cytosolic Ca^2+^. Stimulation of primary neutrophils with either of the two recombinant SAA variants resulted in clear cytosolic Ca^2+^ transients (Figure 7B). In complete agreement with the ROS production data (Figure 7A), the FPR2 antagonist WRW4 completely abrogated the Ca^2+^ signal induced by rSAA1, whereas the signal induced by rSAAh was insensitive to the FPR2 antagonist (Figure 7B). Thus, rSAA1 is indeed a bona fide ligand for FPR2 in human neutrophils. Further, the relatively minor amino acid differences between rSAA1 and rSAAh result in a marked difference in receptor usage, where the receptor utilized by rSAAh remains unidentified.

## Discussion

The systemic acute phase response to inflammation results in dramatic changes in the serum levels of acute phase proteins. These proteins are mainly produced by the liver and are part of an evolutionarily conserved system aimed at controlling infections, eliminating components from damaged tissue, and to protect against inflammation-evoked (collateral) damage. The protein SAA is one of the most notable acute phase reactants and it has been estimated that 2.5–3% of total liver protein synthesis is dedicated for SAA production during acute inflammation (Kisilevsky and Manley, 2012). The concentrations of SAA in circulation may within hours increase 1000-fold during an acute phase response, but may also be chronically elevated in individuals with various inflammatory conditions, including atherosclerosis, obesity and not least rheumatologic diseases (Cunnane et al., 2000). Such sustained high levels of SAA may lead to reactive amyloidosis, a pathological condition characterized by extracellular deposits of SAA in amyloid fibrils that gradually leads to progressive organ failure (Benson et al., 2006). The exact mechanism for how SAA is transferred into an amyloidogenic, i.e., aggregated form, in humans is not known, though several mechanisms have been suggested (Raynes and McAdam, 1991; Ajiro et al., 2006; Noborn et al., 2012).

Aside from the involvement in reactive amyloidosis, the physiological and pathological roles of SAA are somewhat unclear. Early reports seem to favor anti-inflammatory properties of SAA (Benson and Aldo-Benson, 1979, 1982) in line with a general role of acute phase reactants in dampening the inflammatory onslaught that triggers their synthesis. This view rapidly changed by the advent of the rSAAh that in 1994 was shown to function as a chemoattractant for primary human monocytes and neutrophils (Badolato et al., 1994). A few years later the receptor specificity for SAA was determined by showing that an FPR2-transfected cell line displayed chemotactic migration toward rSAAh, identifying rSAAh as the first endogenous ligand for FPR2 (Su et al., 1999). Since then, most work on the effects of SAA on inflammatory cells has been performed with rSAAh and found it to possess a wide variety of pro-inflammatory activities including the capacity to induce IL-8 production (Furlaneto and Campa, 2000; He et al., 2003; Christenson et al., 2008). Investigations using endogenous, native SAA are considerably scarcer. We have earlier shown that SAA-containing fractions, derived from hydrophobic interaction chromatography followed by gel filtration, were able to suppress neutrophil apoptosis (Christenson et al., 2008). On the other hand, another SAA preparation that was >98% pure did not affect cytokine production (Bjorkman et al., 2010) or neutrophil apoptosis (unpublished data). These somewhat incongruent data indicate that the purification of SAA from human plasma is by no means a trivial task and that low contaminating levels of, e.g., other lipoproteins, are likely to complicate interpretation of data. *In vivo*, pure SAA would be a rare occurrence and we therefore believe that experiments carried out with natural, non-purified, material such as SAA-rich plasma are crucial to obtain a true picture of how this protein really functions.

One recent study of melanoma patients has argued that endogenous SAA drives the production of IL-8 (as well as IL-10) in circulating neutrophils (De Santo et al., 2010), and if extrapolated to other inflammatory conditions, this finding should mean that cells derived from other patient groups associated with elevated SAA levels should also display increased IL-8 content. We found no such IL-8 increase in circulation or in neutrophils from inflammatory arthritis patients, but instead there was a notable lack of correlation between SAA levels and circulating levels of inflammatory cytokines in our group of patients (Table 1; Figure 3). These nine patients represent a limited group and the patients were not subjected to the study in terms of a specific diagnosis but rather the fact that they had elevated levels of SAA. In combination with data from the mouse model where the transgenic mice were actually found to express less KC compared to WT mice, we find it clear that SAA does not drive the production of pro-inflammatory cytokines. Thus, the elevated levels of IL-8 reported in neutrophils from melanoma patients, must be explained by a factor other than SAA.

As mentioned, a number and variety of neutrophil receptors have been suggested to recognize rSAAh. The typical activation pattern induced by rSAAh, involving transient Ca^2+^ elevation and ROS production, suggests that a chemoattractant receptor is at least partly responsible for its cellular effects. The FPR2 is one such chemoattractant receptor that has been demonstrated to recognize rSAAh (Su et al., 1999; He et al., 2003; Lee et al., 2006). We have earlier shown that rSAAh can activate cells through FPR2 when the receptor is over-expressed on cell lines, while this particular receptor is not used for activation of the ROS-producing NADPH-oxidase in primary human neutrophils (Bjorkman et al., 2008). Our data described herein support this, but also show that the other recombinant SAA, rSAA1, indeed activates FPR2 on primary human neutrophils. Further proof that the two recombinant SAA proteins activate neutrophils via different receptors can be obtained from desensitization experiments (Bjorkman et al., 2008). Neutrophils desensitized with a high affinity ligand for FPR2, WKYMVm, were still able to respond to rSAAh while the rSAA1 response was clearly reduced (data not shown). It should be noted that we also observed FPR2-independent effects of rSAA1; WRW4 did not significantly attenuate the anti-apoptotic effect of rSAA1 (data not shown).

It is clear that the relatively minor alterations to the amino acid sequence of rSAA1 into rSAAh, changes the receptor preference of the proteins. Such shifts in receptor usage after small variations in the primary sequences are well known for FPR family receptor ligands (Fu et al., 2006). Furthermore, moderate changes in SAA sequence have been shown to significantly alter fibril formation capacity (de Beer et al., 1993). With this in mind, it is vital for recombinant proteins to share the sequence of the original protein in order for biologically meaningful conclusions to be drawn. Hence, rSAA1 should be a better substitute for endogenous SAA since its sequence is identical to human SAA1 (with the exception of the N-terminal methionine, presumably added in order to initiate transcription in the bacterial expression system).

It has recently been debated in *Arthritis & Rheumatism* (van den Brand et al., 2013) whether the use of recombinant molecules is valid for studying the relationship between SAA and joint destruction in inflammatory arthritis (Connolly et al., 2012). In line with the findings by van den Brand et al. (2013), also our data indicate that rSAAh is more potent than rSAA1 *in vitro*. More importantly, our findings (this paper and Bjorkman et al., 2010) demonstrate a fundamental difference in terms of function between the recombinant and endogenous SAAs. The underlying reason for this dissimilarity is yet unknown, but one hypothesis would be a difference in the association to lipids. The SAA proteins are typically associated with high-density lipoprotein (HDL) and such association could potentially abrogate pro-inflammatory activity of endogenous SAA (Magy et al., 2007). It was recently suggested that only lipid-poor SAA (and not HDL-associated) stimulates cytokine production (Kim et al., 2012), and apart from insoluble amyloid aggregates the existence of lipid-free SAA is highly unlikely (Kisilevsky and Manley, 2012). The difference in neutrophil activating potential between endogenous and recombinant SAA could then be explained by a lack of HDL association by the recombinant proteins. However, in our hands (Bjorkman et al., 2010; and this study) recombinant SAA variants maintained activity after incubation in human plasma (healthy or inflammatory), which is a rich source of HDL and other lipids. Since the N-terminal part of SAA is known to determine lipid binding (Patel et al., 1996), one possible determining factor is that the N-terminal methionine needed for bacterial synthesis of the recombinant proteins alters HDL association.

Our results showing that endogenous SAA lacks the pro-inflammatory effects that are seen for the recombinant variants are in many ways similar to the controversy regarding a potential pro-inflammatory effect of C-reactive protein (CRP). Whereas recombinant CRP activates a wide variety of immune cells, evidence of pro-inflammatory activity in the endogenous protein is lacking (reviewed in Ray et al., 2006). It has been shown that recombinant CRP in fact is heavily contaminated by bacterial endotoxin (Pepys et al., 2005) and we cannot completely rule out that contaminating bacterial products contribute to certain effects of the recombinant SAAs (e.g., delay of apoptosis). However, the Ca^2+^ transients and ROS release triggered by the recombinant SAAs are functional features that are certainly not induced by LPS.

We conclude that even though recombinant SAAs can be valuable tools for studying various aspects of neutrophil activation, they are not valid substitutes for mimicking the role of endogenous SAA during an acute phase response.

## Conflict of Interest Statement

The authors declare that the research was conducted in the absence of any commercial or financial relationships that could be construed as a potential conflict of interest.
